# Supplementary material for: Splice-Junction-Based Mapping of Alternative Isoforms in the Human Proteome
Source: Cell Rep. Author manuscript; Available in PMC 2020 Jan 15. (PMC6961840; doi:10.1016/j.celrep.2019.11.026)
Supplement: 2 [file NIHMS1546469-supplement-2.zip › DF1/PXD010154/Lung/Lung_50_PLEKHB1_TALLEANSTPVDSALESPFEEMALVR.pdf]

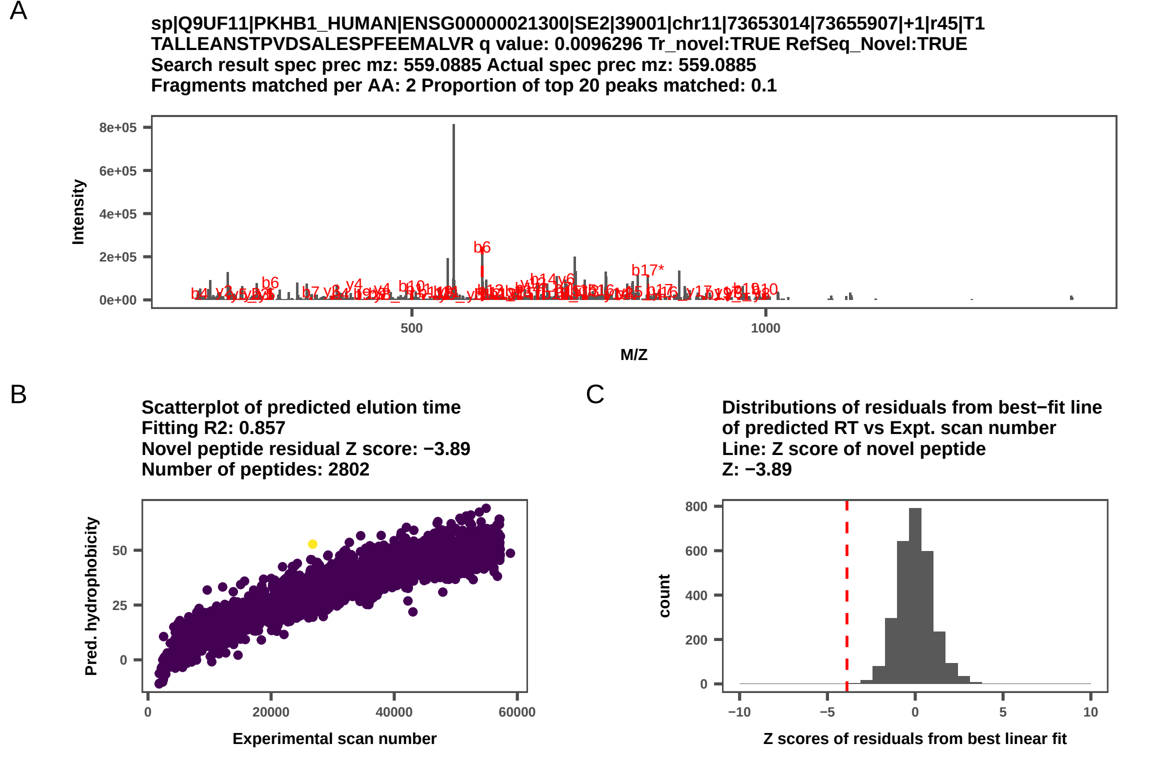
A
sp|Q9UF11|PKHB1_HUMAN|ENSG00000021300|SE2|39001|chr11|73653014|73655907|+1|r45|T1
TALLEANSTPVDSALESPFEEMALVR q value: 0.0096296 Tr_novel:TRUE RefSeq_Novel:TRUE
Search result spec prec mz: 559.0885 Actual spec prec mz: 559.0885
Fragments matched per AA: 2 Proportion of top 20 peaks matched: 0.1
Intensity
8e+05
6e+05
4e+05
2e+05
0e+00
b6
b17*
b14
b10
b6
y4
b7
500
1000
M/Z
B
Scatterplot of predicted elution time
Fitting R2: 0.857
Novel peptide residual Z score: −3.89
Number of peptides: 2802
Pred. hydrophobicity
50
25
0
0
20000
40000
60000
Experimental scan number
C
Distributions of residuals from best−fit line
of predicted RT vs Expt. scan number
Line: Z score of novel peptide
Z: −3.89
count
800
600
400
200
0
−10
−5
0
5
10
Z scores of residuals from best linear fit
